# Perceived Acceptability, Design Preferences, and User Requirements for a Proposed Ear-Worn Device for Heart-Rate and Ear-Temperature Monitoring with Localized Neck Cooling: A Pre-Prototype Mixed-Methods Study in People with Multiple Sclerosis

**DOI:** 10.3390/healthcare14203502

**Published:** 2026-10-18

**Authors:** Afrah Almuwais, Madawi Alotaibi, Dalia Binshaye, Daad Alhumaid, Fatimah Almohsen, Sarah Alsubaie, Rahaf Alshareef, Najd Alkhushayban

**Affiliations:** Department of Rehabilitation Sciences, College of Health and Rehabilitation Sciences, Princess Nourah Bint Abdulrahman University, P.O. Box 84428, Riyadh 11671, Saudi Arabia

**Keywords:** multiple sclerosis, wearable technology, heat sensitivity, ear-worn device, user requirements, acceptability, mixed-methods, pre-prototype

## Abstract

**Background/Objectives:** Heat sensitivity in multiple sclerosis (MS) can worsen neurological symptoms and impair daily functioning and quality of life. As current cooling strategies are largely reactive, wearable technology may offer a proactive approach, provided it is acceptable to users. This pre-prototype study evaluated the perceived acceptability of a proposed ear-worn device, not yet fabricated, that would measure ear-site temperature and heart rate, alert the user, and direct localized airflow at the neck, and identified the user priorities that should guide its development. No technical feasibility or device-performance testing was performed. **Methods:** Adults with MS completed an electronic questionnaire on design preferences, functional features, and overall acceptability; a subset took part in semi-structured interviews analyzed thematically. **Results:** Forty-seven participants completed the survey. Comfort during prolonged use (72.3%) and compatibility with prescription glasses (66.0%) were the design features most often rated important; health reports (93.6%), communication with healthcare providers (91.5%), symptom monitoring (89.4%), emergency contact (85.1%), and automatic cooling activation (83.0%) were each rated important by more than 80% of participants, with overlapping confidence intervals that do not permit these functions to be ranked. Anticipated acceptability of the concept was uncertain: 46.8% (95% confidence interval [CI] 33.3–60.8) expressed some degree of acceptability (34.0% when only ‘acceptable’ or ‘very acceptable’ responses were counted), 36.2% were neutral, and 17.0% found it not acceptable. Six interviews generated four themes: uncertainty, the impact of heat on daily life, independence through self-management, and practical considerations for implementation. **Conclusions:** These findings reflect anticipated, concept-level acceptability rather than acceptability after use of a physical device. Because the proposed device would measure ear-site temperature and heart rate rather than neurological symptoms, the priority given to symptom monitoring is addressed through physiological early warning combined with user-reported symptoms. The identified priorities are translated into measurable specifications and verification tests for the next prototype; the technical performance of the device, including its cooling capacity, remains to be demonstrated.

## 1. Introduction

Multiple sclerosis (MS) is a chronic immune-mediated disease of the central nervous system characterized by inflammation, demyelination, and progressive neuroaxonal loss. It is among the most common non-traumatic causes of neurological disability in young adults, and its estimated global prevalence has risen to approximately 2.8 million people [1]. Prevalence has increased particularly rapidly in the Gulf region, including Saudi Arabia, where a young population and a hot climate coincide [2].

Heat sensitivity is one of the most disabling everyday features of MS. A rise in body temperature of only a few tenths of a degree Celsius can transiently slow or block conduction in demyelinated axons and reproduce or amplify existing symptoms, a phenomenon described by Uhthoff and now understood as one component of a broader thermoregulatory dysfunction in MS [3,4,5]. Uhthoff’s phenomenon is, by definition, transient and reversible: symptoms such as blurred vision, weakness, fatigue or sensory disturbance worsen while core temperature is raised and resolve when the body cools, and they reflect temperature-dependent conduction block rather than new inflammatory disease activity. Such a heat-related pseudo-exacerbation must therefore be distinguished from a true relapse, a distinction that requires clinical assessment [3,4,5]. This dysfunction is not limited to conduction: people with MS show attenuated sweating responses to passive heating and impaired heat loss during exercise, so heat accumulates faster and dissipates more slowly than in unaffected individuals [6,7]. Autonomic dysfunction is also common in MS and can affect cardiovascular control, including heart rate and heart-rate variability, as well as sudomotor function, which further compromises thermoregulation and provides a rationale for monitoring heart rate alongside temperature [8]. Patient-centred surveys show that heat triggers fatigue, weakness, sensory disturbance, visual blurring and cognitive slowing, and that people with MS develop elaborate avoidance routines to remain within a narrow thermal comfort zone [9]. Thermal sensitivity is also regionally heterogeneous across the body surface, which has direct implications for where a cooling stimulus should be applied [10,11].

Cooling is the principal non-pharmacological countermeasure. A sham-controlled trial of cooling garments reported measurable, if modest, improvement in motor and visual function together with persistent subjective benefit [12], and head or neck cooling in particular has been shown to improve exercise tolerance, motor accuracy, and heat-related symptoms in people with MS [13,14,15,16]. The head and neck are attractive cooling targets because of their high thermosensitivity and superficial vasculature [17]. However, cooling vests, cold packs, and air-conditioned environments share three practical limitations: they are bulky or conspicuous, they are applied only after symptoms have been noticed, and they provide no measurement of the physiological change that precedes the symptom.

Wearable sensing has largely been applied in MS to motor function—gait, ambulation, and physical activity—using inertial sensors and smartphones [18,19,20,21]. Thermal status has received far less attention, despite being the trigger that people with MS themselves prioritize. Ear-worn sensing is well suited to this gap. The ear canal and auricle are mechanically stable, comparatively well perfused, and partly shielded from ambient convection, and the ear canal lies close to the tympanic membrane, whose arterial supply is shared with deep cranial structures; this is why tympanic and ear-canal temperatures are widely used as non-invasive surrogates of core temperature. Ear-based devices have been shown to track physiological strain during heat exposure with better agreement when auricular sites are combined [22,23]. Miniaturized infrared thermopile sensing has been demonstrated for non-invasive core-temperature estimation [24], and wearable thermal-strain monitoring frameworks are already established in occupational settings [25,26]. By comparison, wrist and forehead skin temperatures are strongly influenced by ambient conditions, sweating and local vasomotor tone, and chest-worn systems are conspicuous and less suitable for all-day wear [25,26]. Ear-site temperature is nevertheless not identical to core body temperature: it is affected by ambient temperature, airflow, sensor fit and head covering, and its agreement with a reference core-temperature measure must be established for each device [22,23]. An ear-worn platform also places the actuator within centimeters of the lateral neck, allowing sensing and localized cooling to be co-located in a single wearable rather than split across a monitor and a separate cooling garment.

Technical feasibility alone does not determine whether such a system will be used. Acceptability—the extent to which people consider an intervention appropriate, based on their anticipated and experienced cognitive and emotional responses—is a distinct, multi-faceted construct that can and should be assessed before a prototype is finalized [27,28]. Usability reviews of wearables in chronic disease consistently show that comfort, appearance, burden, and perceived usefulness govern sustained wear at least as strongly as measurement accuracy [29], and studies in neurological populations show that the choice to adopt mobile health (mHealth) technology is driven by perceived benefit, data control, and clinical integration [30,31]. Establishing these requirements early is cheaper than discovering them after tooling.

Accordingly, this pre-prototype study had three aims: (i) to describe the proposed sensing and actuation architecture of a patented ear-worn device concept for heat-related symptom management in MS; (ii) to quantify which physical and functional attributes potential users consider important, and how acceptable they anticipate the concept to be overall; and (iii) to convert those user priorities into explicit, measurable engineering specifications and verification tests for the next design iteration. The study did not aim to assess technical feasibility: no device-performance parameters, such as sensor accuracy, photoplethysmography (PPG) performance, temperature-measurement error, response time, cooling capacity, airflow, power consumption, or battery life, were measured.

Terminology. Throughout this article, ‘ear-worn device’ refers to the whole system, which is mounted on the auricle with an arm extending behind the ear; ‘ear-site temperature’ refers to the temperature measured by the device’s sensor at the ear, which is distinct from core body temperature; and ‘localized cooling’ refers to airflow directed at the lateral neck, not to regulation of body temperature. Finally, ‘acceptability’ refers to the anticipated (prospective) acceptability of the described concept, which is distinct from the acceptability experienced after real-world use of a physical device [27].

## 2. Materials and Methods

### 2.1. Study Design and Reporting

This was a sequential mixed-methods study (quantitative-then-qualitative) conducted to establish user requirements and perceived acceptability for the ear-worn system before prototype fabrication; it was not a technical feasibility or device-validation study. A cross-sectional online survey characterized preferences across the whole sample, and semi-structured interviews with a subset of respondents explained the reasoning behind those preferences. Reporting follows the Strengthening the Reporting of Observational Studies in Epidemiology (STROBE) statement for the cross-sectional component [32] and the Consolidated Criteria for Reporting Qualitative Research (COREQ) checklist for the qualitative component [33]; completed checklists are provided as Supplementary Materials.

### 2.2. The Ear-Worn Sensing and Cooling System

**Figure 1:**
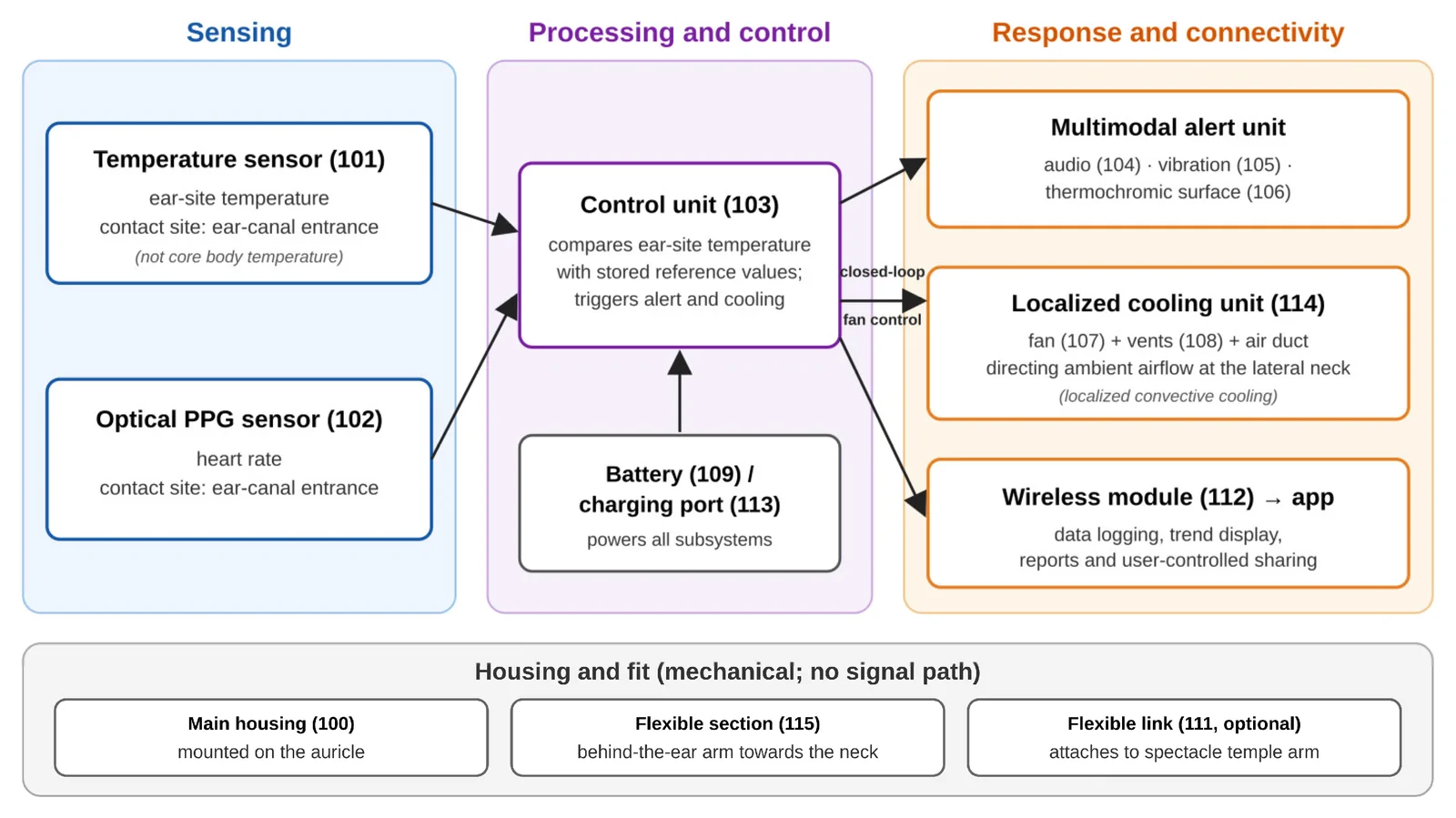
Proposed functional architecture of the ear-worn device (design concept; no functional prototype had been fabricated at the time of the study). Component numbers correspond to the patent specification (SA 1020242964 B1). The temperature sensor (101) measures ear-site temperature, and the optical sensor (102) acquires a photoplethysmographic signal for heart rate, both at the ear-canal entrance; ear-site temperature is not equivalent to core body temperature. The control unit (103) compares ear-site temperature with stored reference values and, on threshold crossing, drives the multimodal alert unit (104–106) and the localized cooling unit (114), whose duct directs ambient airflow at the lateral neck; the control unit also modulates fan speed in a closed loop according to the ongoing thermal reading. The wireless module (112) transmits data to a companion application. The battery (109) powers all subsystems. The housing components (100, 111, 115) are mechanical and carry no signals.

The system evaluated in this study is an ear-worn device concept for continuous physiological monitoring and localized neck cooling, protected by Saudi patent SA 1020242964 B1. Its architecture is summarized in Figure 1; component numbers correspond to those in the patent specification.

*Housing and fit.* A main housing (100) is mounted on the auricle and extends behind the ear towards the lateral cervical region. A flexible section (115) allows the arm to conform to varying ear geometries during movement, and an optional flexible link (111) attaches the housing to the temple arm of prescription or sunglasses to stabilize the device during activity.

*Sensing.* A temperature sensor (101) measures ear-site temperature, and an optical sensor (102) acquires a photoplethysmographic signal from which heart rate is derived. Both are integrated into the inner (skin-facing) aspect of the housing, with the sensing surface in contact with the skin at the entrance of the external ear canal, so that measurements are taken from the ear site continuously during wear. The temperature reading is therefore an ear-site temperature and not a direct measure of core body temperature; its use as a surrogate requires validation against a reference core-temperature measure in prototype testing.

*Processing and control.* An electronic control unit (103) receives the sensor stream and compares it with pre-stored reference values. When the measured ear-site temperature exceeds the stored threshold, the control unit issues an activation signal to the alerting and cooling subsystems. In one embodiment, the control unit modulates cooling intensity continuously as a function of the instantaneous thermal reading, giving closed-loop rather than binary actuation.

*Alerting.* A multimodal alert unit provides redundant notification through an acoustic element (104), a vibration motor (105), and a thermochromic outer surface (106) that changes color with temperature. Redundancy across auditory, tactile, and visual channels is deliberate, since MS commonly involves visual and sensory impairment.

*Localized cooling.* The cooling unit (114) is embedded in the behind-the-ear arm and comprises a fan (107), vent apertures (108), and an air duct oriented inferiorly towards the lateral neck. Cooling is therefore convective–forced ambient airflow over a high-thermosensitivity skin region–rather than conductive or thermoelectric, which avoids the mass, condensation, and power demands of a Peltier stage in an ear-worn form factor.

*Power and connectivity.* A rechargeable internal battery (109) with a charging port (113) supplies the control, alerting and cooling subsystems. A wireless module (112) transmits physiological data to a companion smartphone application for logging, trend display, report generation and sharing.

At the time of this study, the system existed as a protected design concept with defined components and control logic; no functional prototype had been fabricated. Participants therefore evaluated a described and illustrated concept, and the present work is a pre-prototype requirements and acceptability study rather than a device validation study. No technical parameters (sensor accuracy, response time, airflow, cooling capacity, power consumption or battery life) were measured. In the survey, the cooling function was presented as the automatic activation of a cooling system that helps to cool the body (File S1, item 26), and the cooling mechanism (e.g., thermoelectric, evaporative or phase-change) was not specified; in the interviews, it was described as an automatic unit that directs air towards the neck (File S2, question 13).

### 2.3. Participants and Recruitment

Eligible participants were adults aged 18 years or older with a clinically confirmed diagnosis of MS who reported heat sensitivity or symptom worsening associated with a rise in body temperature, and who were able to give informed consent. Individuals under 18 years, those with severe cognitive impairment, and those unable to complete the survey or interview were excluded.

Participants were recruited by convenience sampling through neurology and rehabilitation clinics, MS support groups, and social media; the survey was open from 11 February to 22 June 2026. Of the survey respondents, a subset who indicated willingness to be contacted further was invited to interview.

No a priori sample-size calculation was performed; the survey sample comprised all eligible respondents who completed the survey during the recruitment window. Because the analysis was descriptive, adequacy was judged by precision: with n = 47, the 95% confidence interval (CI) for any proportion has a half-width of at most approximately ±14 percentage points (the worst case, at a proportion of 50%), which was considered sufficient to identify broad priorities but not to separate adjacent features. For the interviews, sample size was guided by the concept of information power rather than by a fixed number [34]: the aim was narrow, participants were highly specific to the aim (heat-sensitive adults with MS who had already completed the survey), and the interviews were in-depth (40–60 min). All respondents who volunteered and could be contacted were interviewed (n = 6).

### 2.4. Questionnaire Development and Content

Due to the absence of a validated instrument for assessing the acceptability of an ear-worn thermal-management device, the research team created a purpose-built questionnaire. This was developed from three key sources: the clinical literature on heat sensitivity in multiple sclerosis (MS) [3,9], the functional specifications of the patented device, and the domains outlined in the Theoretical Framework of Acceptability [27,28]. The questionnaire was administered in Arabic through an online Google Survey. Prior to distribution, its content was reviewed by five experts in neurological rehabilitation, comprising three physical therapists and two occupational therapists (with a mean of 10 years of clinical experience), who judged the relevance, clarity, and comprehensiveness of each item, and appropriate revisions were made based on their feedback. The questionnaire was not adapted from technology-acceptance frameworks such as the Technology Acceptance Model (TAM) or the Unified Theory of Acceptance and Use of Technology (UTAUT), or from usability scales such as the System Usability Scale (SUS), because these assume experience with an existing, working system, whereas participants here evaluated an unbuilt concept. Formal psychometric validation (e.g., content validity index, factor structure, internal consistency or test–retest reliability) was not performed; the instrument should therefore be regarded as a descriptive requirement-elicitation tool rather than a validated measurement scale.

The questionnaire comprised four sections. (i) Demographic and clinical characteristics: age, sex, and time since diagnosis, together with the occurrence, degree, duration, frequency and severity of heat-related symptom worsening, the symptoms experienced, coping strategies, and assistive devices used. MS subtype, disease-modifying treatment and disability level (e.g., Expanded Disability Status Scale [EDSS]) were not collected. (ii) Physical design preferences: three items covering a small and discreet design, comfort during prolonged use, and compatibility with prescription glasses. (iii) Functional-feature priorities: seventeen items grouped a priori, on the basis of the device’s functional architecture rather than of any empirical item structure, into four domains—physiological monitoring, safety and automated response, communication and connectivity, and self-management support. (iv) Overall acceptability: a single item (‘How acceptable do you find the idea of a device worn in the ear?’) rated on a five-point scale whose options, in the order shown on the form, were not acceptable (1), somewhat acceptable (2), neutral (3), acceptable (4) and very acceptable (5). The Theoretical Framework of Acceptability informed the selection of design and feature items (for example, comfort during prolonged use relates to burden, the usefulness of individual functions to perceived effectiveness, and data-sharing preferences to ethicality), but at the concept level only one of its seven constructs, affective attitude, was operationalized, as a single overall rating. Burden, perceived effectiveness, self-efficacy, ethicality, opportunity costs and intervention coherence were not measured as separate constructs, so the overall item is not a measure of acceptability in the multi-construct sense of that framework [27,28].

Design and feature items were rated on a five-point importance scale. For analysis, responses were collapsed into ‘important’ (important/very important), ‘neutral’, and ‘less important’ (not important/slightly important), and are reported as counts and percentages of the full sample. The full instrument is provided as Supplementary Materials.

### 2.5. Semi-Structured Interviews

Semi-structured interviews explored participants’ experience of heat-related symptom worsening, their reaction to the described device, and their recommendations for its development. Survey respondents who agreed to be interviewed (File S1, item 38) were contacted by telephone or email. Interviews were conducted remotely in Arabic by one female author (D.B.), a BSc-qualified researcher who had received training in qualitative interviewing and had no prior relationship with the participants; no one else was present. Interviews lasted 40–60 min, were audio-recorded with consent, transcribed verbatim, and anonymized. No repeat interviews were conducted, field notes were not taken, and transcripts were not returned to participants. An interview topic guide is provided as Supplementary Materials.

### 2.6. Ethical Considerations

Ethical approval was obtained from the Institutional Review Board of Princess Nourah bint Abdulrahman University (IRB log number 26-0259, approved on 9 February 2026). Prior to participation, all participants provided electronic informed consent. Data were anonymized during transcription and securely stored on password-protected devices, accessible only to the research team. The study adhered to the principles outlined in the Declaration of Helsinki.

### 2.7. Data Analysis

Survey data were analyzed in IBM SPSS Statistics version 31 (IBM Corp., Armonk, NY, USA). Descriptive statistics were used to summarize participant demographic and clinical characteristics, physical design preferences, preferred functional features, and overall acceptability of the proposed device. Continuous variables were summarized as mean and standard deviation (SD) or, for skewed data, median and interquartile range (IQR), and categorical variables as counts and percentages, with 95% confidence intervals for proportions calculated using the Wilson score method. Features are presented in descending order of the proportion rating them important for readability only; because the study was not designed to compare features, differences between features with overlapping confidence intervals were not interpreted as differences in priority. To examine whether the a priori grouping of the 17 functional-feature items was supported by the data, Spearman inter-item correlations were calculated from the original five-point ratings, and the mean inter-item correlation and Cronbach’s alpha were computed for each domain and compared with the mean correlation between items from different domains. Exploratory factor analysis was not performed: with 47 respondents for 17 items (fewer than three respondents per item), the sample is well below the minimums commonly recommended for factor analysis, and any factor solution would be unstable and potentially misleading [35,36]. These analyses were exploratory and were not intended as a psychometric validation of the instrument. The characteristics of the interview participants were summarized descriptively and compared with those of the full survey sample. Because the survey form placed ‘somewhat acceptable’ below ‘neutral’, overall acceptability is reported under two definitions: a broad definition (very acceptable, acceptable or somewhat acceptable) and a strict definition (very acceptable or acceptable only). No inferential or subgroup analyses were performed, because the study was not powered for them.

Interview transcripts were analyzed using reflexive thematic analysis following the six phases described by Braun and Clarke [37]: familiarization, initial coding, searching for themes, reviewing themes, defining and naming themes, and reporting. Coding was inductive and semantic and was performed manually in Microsoft Word. All six transcripts were coded independently by two authors (DB and MA), who then compared their codes; discrepancies were resolved by discussion until consensus was reached, and candidate themes were reviewed by the wider research team. Consistent with the reflexive approach, in which coding is an interpretive rather than a measurement process, inter-rater agreement statistics were not calculated [38]. Thematic saturation was not formally assessed; the adequacy of the interview sample was judged using information power (Section 2.3) [34]. Quantitative and qualitative findings were integrated at the interpretation stage using a joint display, in which each prioritized feature was mapped to the interview accounts that explained its importance.

## 3. Results

### 3.1. Participant Characteristics

**Figure 2:**
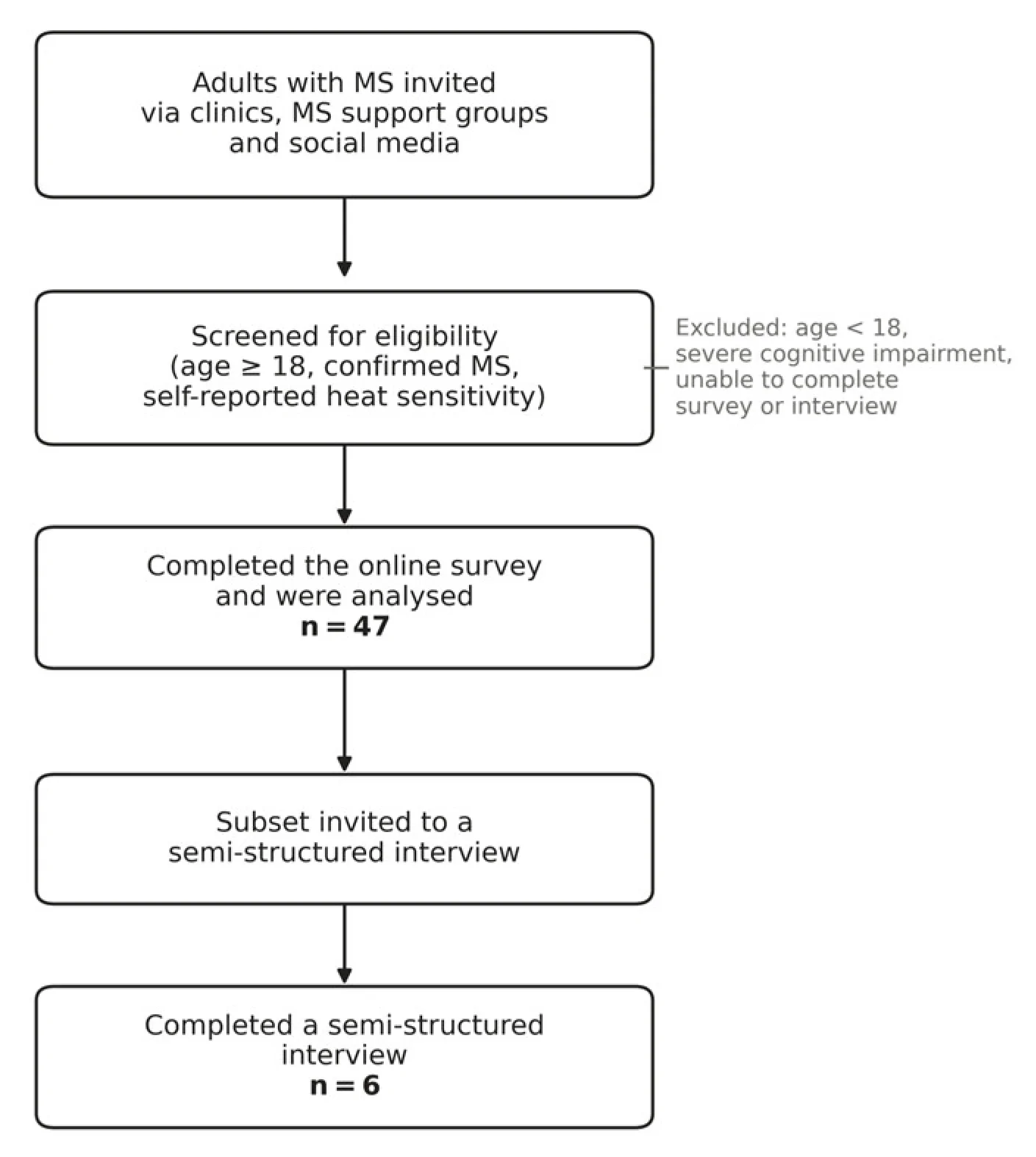
Participant flow through the survey and interview components of the study.

A total of forty-seven participants (Figure 2) completed the survey and were included in the analysis. The mean age of the participants was 35.6 ± 7.9 years, with 29 individuals (61.7%) identifying as female and 18 (38.3%) as male. The median time since diagnosis was 9 years (IQR 4–17; n = 45 respondents who gave an interpretable answer to this free-text item). The demographic and clinical characteristics are detailed in Table 1. MS subtype, disease-modifying treatment, and disability level were not collected (Section 2.4). Self-reported heat-related symptom worsening, described below, is reported as the indicator of a history of Uhthoff’s phenomenon; it was not clinically verified. All demographic and clinical variables collected in the survey are reported in Table 1, and Table S3 presents the same characteristics for the six interview participants alongside those of the full sample.

Worsening of heat-related symptoms was nearly universal, with 25 participants (53.2%, 95% CI 39.2–66.7) reporting that an increase in body temperature exacerbated their symptoms. Additionally, 15 participants (31.9%) indicated that this occurred occasionally, resulting in 40 out of 47 participants (85.1%, 72.3–92.6) experiencing heat-related symptom worsening at least intermittently. The intensity of this effect was rated as moderate by 18 participants (38.3%) and large by 16 participants (34.0%), while 10 of the 47 participants (21.3%) reported a mild effect.

In terms of the typical duration of an attack, among the participants who responded to this question (n = 41), more than half indicated that their attacks lasted longer than 24 h (n = 22, 53.7%). Comparatively, 11 participants (26.8%) reported that their attacks lasted less than 24 h, and 8 participants (19.5%) stated that their attacks lasted exactly 24 h. Regarding the intensity of the attacks, the majority of participants described them as moderate (n = 24, 51.1%), followed by severe (n = 13, 27.7%) and mild (n = 10, 21.3%).

### 3.2. Physical Design Preferences

**Figure 3:**
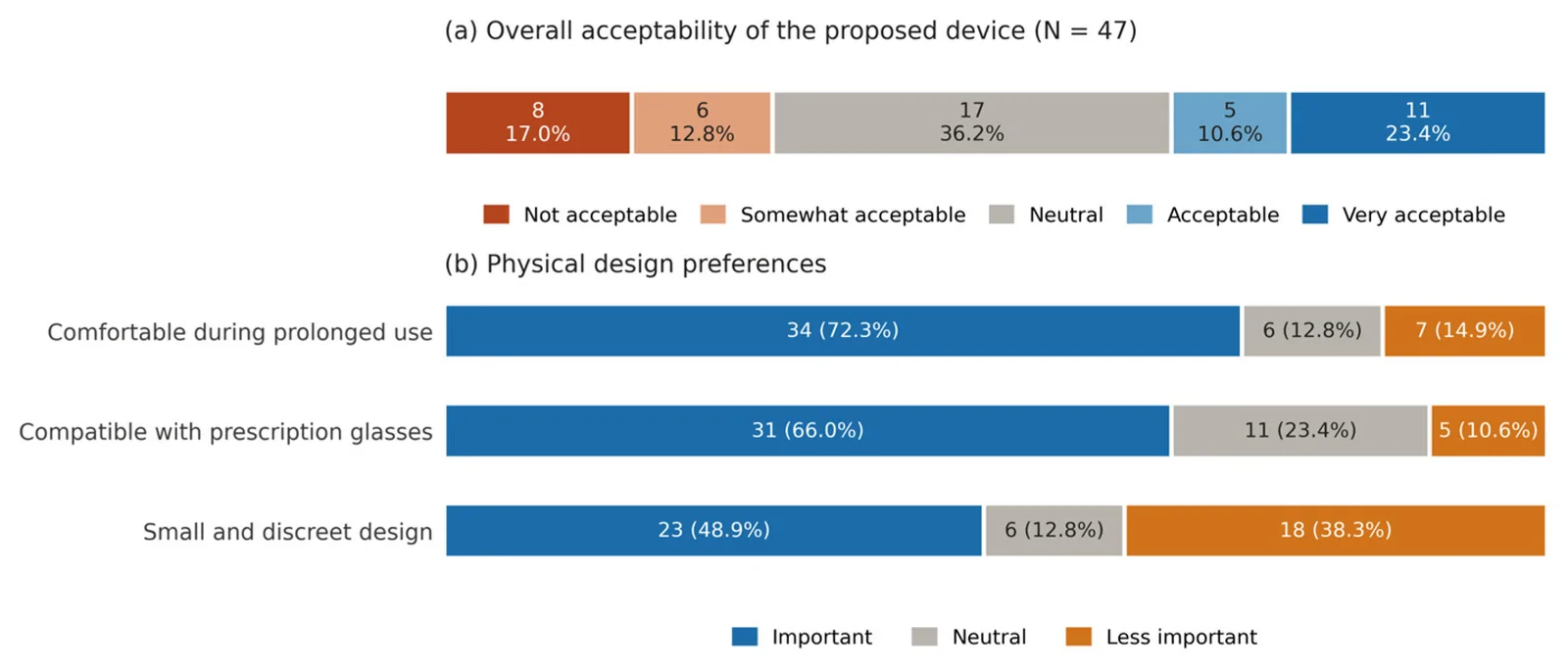
(**a**) Distribution of overall acceptability ratings for the proposed device (n = 47). (**b**) Physical design preferences. Counts and percentages are shown within each segment.

Physical design preferences are shown in Table 2 and Figure 3b. Comfort during prolonged use (34, 72.3%, 58.2–83.1) and compatibility with prescription glasses (31, 66.0%, 51.7–77.8) were each rated important by about two-thirds or more of participants, with overlapping confidence intervals. Opinion on a small and discreet design was divided: 23 participants (48.9%, 35.3–62.8) rated it important, while 18 (38.3%) rated it less important–the only physical attribute on which the sample was not broadly aligned.

### 3.3. Preferred Functional Features

Functional-feature priorities are shown in Table 3. Within physiological monitoring, monitoring of symptom worsening was rated important by 42 participants (89.4%, 77.4–95.4); this survey item describes a desired function, whereas the proposed device would measure ear-site temperature and heart rate rather than symptoms themselves (Section 4.2), heart-rate monitoring by 36 (76.6%), continuous temperature display by 35 (74.5%), and temperature monitoring itself by 32 (68.1%, 53.8–79.6); the confidence intervals of all four items overlapped.

In the safety and automated response domain, emergency contact during an attack (n = 40, 85.1%, 72.3–92.6), automatic cooling activation (n = 39, 83.0%, 69.9–91.1), and immediate multimodal alerting (n = 37, 78.7%, 65.1–88.0) were all prioritized by more than three-quarters of the sample.

For communication and connectivity, communication with a healthcare provider (n = 43, 91.5%, 80.1–96.6) and an integrated health application (n = 41, 87.2%, 74.8–94.0) were rated important by similar proportions of participants. Family access to health status was rated important by the smallest proportion of participants (n = 28, 59.6%, 45.3–72.4), with 14 (29.8%) rating it less important; its confidence interval did not overlap with those of the four features rated important by about 90% of participants.

Within self-management support, health reports (n = 44, 93.6%, 82.8–97.8), physical activity tracking (n = 42, 89.4%), symptom recording (n = 41, 87.2%), medication reminders (n = 40, 85.1%), a daily symptom diary (n = 38, 80.9%), physiological summaries (n = 36, 76.6%) and daily summaries (n = 35, 74.5%) were each rated important by three-quarters or more of participants. It is worth noting that the confidence intervals of the highest- and lowest-ranked features within this domain overlap substantially; the ranking should be read as a gradient of emphasis rather than as a set of statistically separable priorities.

Across domains, health reports, communication with a healthcare provider, monitoring of symptom worsening and physical activity tracking were rated important by approximately 90% of participants (89.4–93.6%), with overlapping confidence intervals; the data therefore do not allow these features to be distinguished from one another, and the order of features in Table 3 is descriptive only.

Inter-item correlations among the 17 functional-feature items are reported in Table S4. The mean within-domain correlation was 0.61 for physiological monitoring, 0.39 for safety and automated response, 0.30 for communication and connectivity, and 0.56 for self-management support, compared with a mean between-domain correlation of 0.39; Cronbach’s alpha was 0.86, 0.60, 0.42 and 0.85, respectively. The data therefore supported the physiological-monitoring and self-management groupings but not the safety and communication groupings: family access to health status was almost uncorrelated with every other item (ρ = −0.12 to 0.22), and monitoring of symptom worsening correlated more strongly with communication with a healthcare provider, the integrated health application and health reports (ρ = 0.71–0.79) than with the temperature and heart-rate items (ρ = 0.41–0.52). The four domains should therefore be regarded as content-based groupings rather than empirically distinct dimensions.

### 3.4. Overall Acceptability

Overall acceptability is shown in Table 4 and Figure 3a. Eleven participants (23.4%) rated the concept very acceptable, 5 (10.6%) acceptable, and 6 (12.8%) somewhat acceptable, giving 22 of 47 (46.8%, 33.3–60.8) expressing some degree of acceptability. Seventeen participants (36.2%, 24.0–50.5) were neutral, and 8 (17.0%, 8.9–30.1) rated the concept not acceptable. Because the survey form placed ‘somewhat acceptable’ below ‘neutral’ (File S1, item 16), a stricter definition counting only ‘acceptable’ or ‘very acceptable’ responses gives 16 of 47 (34.0%, 22.2–48.3). Under either definition, fewer than half of participants expressed positive acceptability, and 25 (53.2%, 39.2–66.7) were neutral or rated the concept not acceptable. Given the width of these confidence intervals and the size of the neutral group, anticipated acceptability of the concept is best characterized as uncertain.

The survey did not collect disability level, prior use of cooling garments, or the presence of tactile allodynia, and the number of participants who rated the concept not acceptable (n = 8) was too small for meaningful subgroup comparison. Differences between accepting and non-accepting respondents were therefore not analyzed statistically; possible reasons for hesitancy are explored qualitatively (Section 3.5, Theme 4) and in Section 4.3.

### 3.5. Interview Findings

Six participants were interviewed. Four themes were generated, summarized below with illustrative quotations. Given the small number of interviews, these themes are presented as an explanatory complement to the survey and may not capture the full range of perspectives in the wider MS population (Section 4.6). Table S3 compares the characteristics of the six interviewees with those of the full survey sample. The interviewees were five women and one man aged 29–43 years (mean 36.7 ± 6.4), with a median time since diagnosis of 13.5 years (range 5–27). In the survey, one had rated the concept very acceptable, one somewhat acceptable, two neutral, and two not acceptable, so participants who did not accept the concept were represented. Compared with the full sample, the interview subgroup included proportionally more women, had a longer time since diagnosis, and more often reported severe episodes (3 of 6 versus 27.7%). For each theme, the number of participants whose accounts contributed to it is reported, and two or more quotations from different participants are given.

#### 3.5.1. Theme 1. Living with Uncertainty

Participants described uncertainty as a constant feature of living with MS: delayed recognition of symptoms, difficulty attributing new symptoms to MS, and, most consistently, difficulty distinguishing a true relapse from a transient pseudo-exacerbation. This uncertainty shaped decisions about whether to rest, continue an activity, or seek medical assessment, and was itself a source of anxiety. Participants also noted that fatigue, cognitive difficulty, and weakness are invisible to others, which compounded misunderstanding of their condition. This theme was expressed by five of the six participants.

“Sometimes I don’t know whether it’s a real attack or a pseudo-attack.”

(Participant 3)

“My whole right side was numb. I went to the clinic and described what I had, and they told me it was a psychological condition.”

(Participant 1)

“What bothers me most is that my family, my wife or those closest to me do not believe me.”

(Participant 4)

#### 3.5.2. Theme 2. The Impact of Heat on Daily Life

Heat was described consistently as a factor that worsened symptoms and constrained activity, producing fatigue, dizziness, weakness, and reduced mobility. Participants restructured their routines around it–avoiding outdoor activity, remaining in air-conditioned spaces, and using cold water or cold packs. The consequences extended beyond physical symptoms to work, volunteering, shopping, travel and social participation. One participant reported being more affected by cold than by heat, but the dominant pattern was that heat sensitivity was a major barrier to everyday functioning. This theme was expressed by five of the six participants.

“When I feel hot, I can’t continue what I’m doing. I have to stop and cool down.”

(Participant 2)

“If it is very hot, I try not to go out; if I feel the heat, I switch on the air conditioning and take a cold shower.”

(Participant 6)

“If a place is hot, it is fine, but the cold affects me.”

(Participant 5; contrasting view)

#### 3.5.3. Theme 3. Supporting Independence Through Self-Management

Participants wanted tools that preserved independence rather than creating dependence on others. What they valued was advance information: enough warning to reorganize a day, rest, or decide whether clinical assessment was warranted. They also valued longitudinal records that could be shared with clinicians, and suggested decision-support features such as graded warning levels, symptom checklists, and help in distinguishing a relapse from a pseudo-exacerbation. This theme was expressed by four of the six participants.

“If I knew earlier that my symptoms were getting worse, I could organize my day and avoid making things worse.”

(Participant 3)

“I want it to alert me, and then I choose whether it intervenes or I intervene myself.”

(Participant 2)

“Honestly, that would be an achievement; this is what we wish for.”

(Participant 1; on an application through which the physician could follow the condition remotely)

#### 3.5.4. Theme 4. Practical Considerations for Implementation

Acceptance was conditional on practicalities. Comfort, low mass, discreet appearance and simple operation were repeatedly raised as prerequisites for daily wear, alongside reliable measurement and cooling that is actually felt. Views on data sharing diverged: sharing with clinicians was broadly welcomed, whereas sharing with family was something participants wanted to control themselves. Participants also asked for compatibility with everyday items worn on or near the head, including prescription glasses and hijab. This theme was expressed by five of the six participants.

“Cooling is more important than just receiving a warning.”

(Participant 2)

“The problem is if it looks like AirPods… it should be shaped so that it does not show that the person has MS.”

(Participant 3)

“I don’t know, honestly. Maybe on the hand or the leg, because MS is not just one type.”

(Participant 5; on placement in the ear)

## 4. Discussion

This study established user requirements and anticipated acceptability for a proposed ear-worn system that would combine continuous ear-site temperature and heart-rate sensing with threshold-triggered convective cooling of the lateral neck, before a prototype was built. Three findings stand out. First, heat-related symptom worsening was reported at least intermittently by 85.1% of participants and rated moderate or large by 72.3%, confirming that the problem the system addresses is prevalent in this sample. Second, the interpretive and communicative functions of the system—health reports (93.6%), communication with a healthcare provider (91.5%), and monitoring of symptom worsening (89.4%), which the system would address indirectly through ear-site temperature and heart-rate trends combined with user-reported symptoms (Section 4.2)—were each rated important by approximately 90% of participants, a proportion at least as high as for the sensing and cooling functions themselves; because the confidence intervals overlap, these features cannot be ranked against one another. Third, and in apparent tension with the second, overall anticipated acceptability of the concept was uncertain: 46.8% expressed some acceptability (34.0% under the strict definition) while 36.2% remained neutral. Taken together, these findings suggest that the value proposition of the device, as perceived by users, is not primarily ‘a thermometer with a fan’ but a decision-support system whose sensing and cooling are the means rather than the end. Here, ‘decision support’ means structured information that helps users and clinicians make decisions; it does not mean automated diagnosis. Because no prototype was built or tested, these findings do not demonstrate the technical feasibility of the device.

### 4.1. The Burden of Heat Sensitivity Supports the Design Premise

The burden of heat sensitivity observed here is consistent with the wider literature. Uhthoff’s phenomenon and the broader thermoregulatory dysfunction of MS mean that small increases in body temperature can transiently degrade conduction in demyelinated axons [3,4,5], and impaired sweating and heat dissipation make such increases both more likely and more persistent [6,7]. Our participants’ accounts—fatigue, dizziness, heaviness, reduced mobility, curtailed work and social participation—mirror the patient-centred findings of Christogianni et al., in which people with MS describe organizing daily life around thermal avoidance [9]. That more than half of participants reported episodes lasting longer than 24 h is notable for design: an actuator intended to shorten or blunt such episodes must be wearable for hours, not minutes, which places the power budget and comfort requirements at the centre of the engineering problem rather than at its periphery.

### 4.2. From User Priorities to Sensing and Actuation Requirements

The primary contribution of this study to device development lies in its ability to translate stated preferences into actionable specifications. Table 5 outlines this mapping. Two examples highlight the underlying logic. More participants rated ‘monitoring of symptom worsening’ important (89.4%) than ‘body-temperature monitoring’ (68.1%), although the confidence intervals overlapped slightly. Symptom worsening, however, cannot be detected by a temperature sensor: a change in ear-site temperature is a physiological trigger, whereas symptom worsening is a clinical outcome that the person experiences and reports, and the two are not the same thing. We therefore interpret this preference as two separate requirements. First, on the sensing side, the system should detect changes in ear-site temperature relative to the individual’s own baseline rather than against a fixed absolute threshold, which requires attention to thermal resolution, drift stability, and per-user calibration rather than to absolute accuracy alone. Second, symptom worsening itself must be captured through user input, for example through the symptom-recording and daily-diary functions that participants also rated highly (87.2% and 80.9%), so that temperature changes and reported symptoms can be viewed together. Whether changes in ear-site temperature predict symptom worsening in a given individual is an empirical question for future studies, not an assumption of the design.

It should also be stated explicitly that temperature and heart-rate measurements alone cannot diagnose an MS relapse or reliably distinguish a relapse from a heat-related pseudo-exacerbation. Although interviewees asked for help with this distinction (Theme 3), any decision-support function addressing it would require separate clinical development and validation, would at most prompt the user to seek clinical advice, and should not replace clinical assessment.

Ear-based measurement presents distinct advantages, as the measurement site is partly shielded and well-perfused. Furthermore, research indicates that combining canal and auricular measurements enhances alignment with physiological strain during heat exposure [22,23]. Additionally, miniaturized infrared thermopile sensing has been proven effective for non-invasive core-temperature estimation [24], while established frameworks for thermal-strain monitoring offer a model for converting a raw thermal signal into a practical index [25,26]. Nevertheless, the proposed device measures ear-site temperature, not core body temperature, and the two should not be conflated; ear-site readings are affected by ambient temperature, wind, sensor fit and head covering, and their agreement with a reference core-temperature measure must be demonstrated before the signal can be interpreted as a surrogate of core temperature.

The second example focuses on cooling. Automatic activation of cooling systems was deemed a priority by 83.0% of participants, with one interviewee asserting that cooling is more important than warnings. There is a substantial evidence base supporting head and neck cooling in multiple sclerosis: sham-controlled and crossover studies indicate improvements in exercise tolerance, motor accuracy, fatigue, and heat-related symptoms [12,13,14,15,16]. Additionally, the neck is an area characterized by high thermosensitivity and superficial vasculature [17], making it an effective target for cooling interventions. What the current findings contribute is a specification regarding dosage: forced convective airflow from an ear-mounted duct must lead to a noticeable change in local skin temperature and thermal sensation that the user can actually perceive. If not, the feature will be considered ineffective, irrespective of its physiological benefits. Therefore, quantifying changes in local skin temperature, airflow velocity at the neck, and thermal sensation ratings should be prioritized as primary endpoints in both initial bench testing and human factors evaluations, rather than being relegated to secondary status. The present study did not measure cooling performance, and it should not be inferred that the proposed device regulates body temperature: its intended function is localized cooling of the neck to relieve heat-related discomfort and symptoms, and whether it produces a measurable change in local skin temperature, thermal sensation or core temperature remains to be tested.

A further constraint is thermodynamic. Because the fan moves ambient air, convective heat exchange at the neck depends on the difference between skin temperature (typically about 33–35 °C) and air temperature. When ambient air approaches or exceeds skin temperature, as is common outdoors during Saudi summers, when shade air temperatures frequently exceed 40 °C, forced airflow may add heat to the skin rather than remove it, and any remaining benefit depends on the evaporation of sweat, which is itself impaired in many people with MS [6,7]. Studies of electric fans in extreme heat indicate that fan use can increase heat strain at very high air temperatures, particularly in dry heat or when sweating is limited [39,40]. Purely ambient-air convective cooling is therefore best suited to indoor, shaded, or transitional environments and to use before or after heat exposure. For extreme outdoor conditions, the next prototype should include an ambient-temperature sensor that suspends or limits fan operation when air temperature approaches skin temperature, and alternative or hybrid cooling (e.g., thermoelectric, evaporative or phase-change elements) should be evaluated against its added mass, condensation and power costs (Table 5).

The engineering trade-offs of ear-level cooling must also be acknowledged. The fan, duct and battery add mass to the device, and mass placed behind the auricle creates a movement of the ear that increases contact pressure and displacement during head movement; for this reason, Table 5 specifies centre-of-mass placement and contact-pressure mapping. Unlike a thermoelectric (Peltier) element, whose rejected heat must be dissipated close to the skin, a fan does not create a hot side, but the motor, electronics, and battery still generate heat within the housing. The fan will also dominate the power budget, and the battery capacity that fits in an ear-worn housing is small, of the order of that in hearing aids or wireless earbuds, so continuous fan operation over a hot day is unlikely. Duty-cycled, threshold-triggered operation and an optional external power source (for example, a neck-worn battery) should therefore be explored and verified by runtime measurement.

### 4.3. Interpreting Uncertain Acceptability

The disparity between high feature-level ratings and uncertain concept-level acceptability warrants careful interpretation rather than overly optimistic reporting. Within the framework of acceptability theory, our single global item reflects an overall affective attitude but does not encompass other important constructs such as burden, perceived effectiveness, self-efficacy, ethicality, opportunity cost, and intervention coherence [27,28]. There is therefore a mismatch between the theoretical framework that informed the questionnaire and the way acceptability was operationalized: the study captured anticipated affective attitude towards a concept, not acceptability in the full multi-construct sense of the framework. A participant may find each function desirable while still harboring doubts about whether an unbuilt device will be comfortable, effective, discreet, or reliable enough for daily use; this uncertainty may manifest as a neutral response. Consequently, the 36.2% neutral response rate should be viewed as an indication of unresolved uncertainty regarding an unbuilt artifact rather than as tacit approval; it may equally reflect lack of interest in the concept or an inability to form a well-founded judgement about a device that does not yet physically exist, and these possibilities cannot be distinguished with the present data, while the 17.0% of respondents who rated the concept as unacceptable represents a noteworthy minority that should be explored in future research rather than dismissed.

Two distinct sources of hesitancy emerge from the data. The mixed opinions on a small and discreet design (48.9% identifying it as important, while 38.3% deem it less so) indicate that the concept of visibility holds different implications for various users—some experience stigma, while others view it as a valid trade-off for comfort and battery life. This underscores the necessity for a form factor decision driven by user segmentation rather than simply relying on the average response. Similarly, the importance placed on family access to health status was the lowest-rated feature (59.6%), whereas clinician communication received one of the highest ratings (91.5%). Hence, data sharing is not a uniform preference but rather directional. The design implication is clear: sharing should be granular and set to off by default, with consent managed on a per-recipient basis under the user’s control. This aligns with findings from neurological populations that indicate data control significantly influences mHealth adoption, as well as usability reviews highlighting that comfort, burden, and perceived usefulness are critical factors for sustained use [29,30,31].

The acceptability estimate itself should not be overstated. Only 34.0% of participants rated the concept acceptable or very acceptable, and more than half (53.2%) were neutral or rated it not acceptable. Several MS-specific barriers may explain this. Upper-limb weakness, ataxia, tremor or reduced finger sensation can make it difficult to insert, position and remove a small ear-worn device and to operate its controls and charging port. A device in or at the ear canal may partly occlude hearing and reduce awareness of environmental sounds, and it may become uncomfortable over several hours. Aesthetic concerns and the stigma of a visible medical device, the bulk added by a battery and fan, and compatibility with glasses and hijab were also raised in the interviews (Theme 4). In addition, the acceptability item asked specifically about ‘a device worn in the ear’, so part of the hesitancy may relate to ear placement itself rather than to the functions offered. Because the survey did not measure these factors, their contribution cannot be quantified here; they should be assessed directly in human-factors testing with a multi-construct acceptability instrument.

### 4.4. Interpretive Caveats

Three caveats govern the interpretation of these findings. First, the ordering of features in Table 2 and Table 3 is purely descriptive. Health reports, communication with a healthcare provider, monitoring of symptom worsening, and physical activity tracking were each rated important by approximately 90% of participants, and their confidence intervals overlap; the study therefore does not permit differentiation among the features in this most highly rated group, and a statement that any one of them was the most valued would not be supported. Only larger differences, such as that between these features and family access to health status, are likely to reflect genuine differences in priority. Second, overall acceptability should be characterized as uncertain rather than moderate: the broad estimate of 46.8% has a 95% confidence interval of 33.3% to 60.8%, which is compatible with acceptability among either a minority or a majority of the population, and the strict estimate is lower still (34.0%). The neutral group (36.2%) is the key interpretive benchmark, and it indicates uncertainty about a device that does not yet exist rather than implicit approval. Third, all ratings refer to the anticipated acceptability of a described and illustrated concept. Anticipated acceptability is an unreliable surrogate for acceptability after use, particularly for a device whose perceived value depends on tactile experience (the sensation of airflow cooling), auditory experience (alert signals and fan noise), and comfort during prolonged wear, none of which can be conveyed by a video, an illustration, or an interview. The present findings therefore show interest in, and perceptions of, the concept at the pre-prototype stage; they do not show whether a physical device would be acceptable in real-world use, which must be established in prototype testing [27,28].

### 4.5. Relation to Existing Cooling and Monitoring Approaches

Compared with existing approaches, the proposed system is distinguished less by any single component than by their co-location. Cooling garments deliver cooling without sensing; wearable monitors in MS deliver sensing, overwhelmingly of motor function, without actuation [18,19,20,21]. A device that measures at the ear and acts on the neck closes that loop within one wearable, which is the specific gap the patent addresses. This advantage is, at present, an argument from architecture rather than from evidence: no comparative data exist, and the claim that co-located sensing and cooling outperforms a monitor plus a separate cooling garment remains to be tested.

### 4.6. Limitations

Several limitations bound these conclusions. First, no functional prototype was built or tested, so the study provides no evidence of technical feasibility, sensor accuracy, cooling capacity or battery life. Participants were recruited by convenience sampling through clinics, support groups and social media and completed an electronic survey, which is likely to over-represent people who are engaged with technology and motivated by heat sensitivity, and the direction of this bias is towards over-estimating acceptability. The sample of 47 yields wide confidence intervals—the interval for overall acceptability spans 33.3% to 60.8%—so the ranking of adjacent features should not be over-interpreted; no a priori sample-size calculation was performed. Participants evaluated a described concept rather than a working prototype, so the findings represent anticipated rather than experienced acceptability, not acceptability after prolonged physical wear in real-world thermal environments, and hypothetical evaluations of unbuilt technology are known to shift once a device is worn. This limitation is substantial: the tactile, auditory, and comfort experiences on which the value of the device depends could not be evaluated, and the neutral responses may reflect lack of interest or an inability to judge an unbuilt device as much as uncertainty. Acceptability was measured with a single unvalidated global item rather than a multi-construct instrument, and because the form placed ‘somewhat acceptable’ below ‘neutral’, the broad estimate (46.8%) should be read alongside the strict estimate (34.0%). The questionnaire was purpose-built, and its measurement properties were not formally established. Exploratory factor analysis was not feasible with 47 respondents, and the a priori grouping of feature items was examined only through inter-item correlations (Table S4). The qualitative component comprised only six interviews with volunteers from the survey, and thematic saturation was not formally assessed, and the themes may disproportionately reflect the views of particularly articulate or motivated participants. With so small a sample, the perspectives of people with greater disability, older people, and those who rated the concept not acceptable may be under-represented, which may have limited the depth and breadth of the four themes; the themes should therefore be read as an explanatory complement to the survey rather than as a comprehensive account. Finally, MS subtype, disease-modifying treatment and disability level (e.g., EDSS) were not collected, and time since diagnosis was collected only as a free-text item; a history of Uhthoff’s phenomenon was self-reported rather than clinically verified; this limits assessment of how far these preferences generalize across the MS population, particularly because disability and upper-limb function are likely to affect the ability to handle an ear-worn device.

### 4.7. Future Work

The immediate priority is the fabrication and bench characterization of a prototype in accordance with the specifications outlined in Table 5. This includes assessing the thermal resolution, drift, and response time of the ear-site sensor, ensuring that the derived thermal index aligns with a reference core-temperature measurement through Bland–Altman analysis, and verifying the agreement of PPG heart rates with electrocardiography during both rest and movement. Additionally, we will evaluate airflow velocity, local neck skin temperature changes, and the acoustic noise produced by the cooling unit, alongside the power budget and runtime during realistic duty cycles, as well as ensuring skin-contact safety during prolonged wear. Cooling performance will be tested in a climatic chamber across ambient temperatures up to 45 °C to define the conditions under which ambient-air cooling is effective. Future studies should also record MS subtype, disability level (EDSS) and upper-limb function.

Following this, human-factors testing will focus on evaluating comfort and retention during extended wear periods, alert detectability, and the usability of the companion application for both users and clinicians. Only after these evaluations should a controlled study be conducted to address the primary clinical question motivating the design: whether early warning based on ear-site temperature and localized neck cooling can reduce the frequency, duration, or severity of heat-related symptom exacerbation compared to a sham-airflow control. Secondary outcomes to be assessed include fatigue, participation in activities, and quality of life. Acceptability should then be re-evaluated at this stage using a multi-construct instrument, such as the theory-informed questionnaire based on the Theoretical Framework of Acceptability [28], in a sample large enough for psychometric evaluation, as the acceptability of a worn device differs from the acceptability of its description.

## 5. Conclusions

In this pre-prototype study, adults with MS identified both the challenges they face and the characteristics of an acceptable solution: heat-related symptom exacerbation was both prevalent and disruptive. Participants emphasized the need for early warning of rises in ear-site temperature and heart rate that may precede heat-related symptom worsening, together with user recording of symptoms, automatic localized cooling, and effective reporting and clinician-communication functions that facilitate decision-making based on measurements. Overall anticipated acceptability of the concept was uncertain (46.8%, 95% CI 33.3–60.8, under a broad definition and 34.0% under a strict definition), with more than a third of respondents remaining neutral. This reflects uncertainty about a device that has yet to be constructed, rather than tacit approval, and highlights the specific areas where prototype development must excel: providing demonstrable comfort over extended wear, compatibility with glasses and headgear, effective cooling sensations, and user-controlled data sharing. This work’s contribution lies in establishing these user priorities as measurable engineering specifications prior to committing to hardware development, ensuring that the first prototype of this ear-worn sensing and cooling system is evaluated against the actual needs expressed by its intended users. Because no prototype was built or tested, these findings do not demonstrate the technical feasibility of the device or its ability to regulate body temperature, and ear-site temperature and heart rate alone cannot identify an MS relapse; the findings define the requirements against which the prototype must now be tested. These results describe anticipated, concept-level acceptability; acceptability after real-world use of a physical prototype remains to be established.

## 6. Patents

The device described in this article is protected by Saudi Arabian patent SA 1020242964 B1, ‘In-ear wearable device to detect body temperature and heart rate and control body temperature’, granted 25 June 2026, on which Madawi Alotaibi, Afrah Almuwais, Dalia Binshaye, Daad Alhumaid, Fatimah Almohsen, Rahaf Alshareef, Najd Alkushiban and Sarah Alsubaie are named inventors.

## Figures and Tables

**Table 1:** Participant demographic and clinical characteristics (n = 47).

Characteristic	Category	n (%) or Mean ± SD
Age (years)	Mean ± SD	35.6 ± 7.9
Sex	Male	18 (38.3)
	Female	29 (61.7)
Time since diagnosis (years), n = 45	Median (IQR)	9 (4–17)
Heat-related symptom worsening	Yes	25 (53.2)
	Sometimes	15 (31.9)
Degree of effect of raised body temperature	No effect	3 (6.4)
	Mild	10 (21.3)
	Moderate	18 (38.3)
	Large	16 (34.0)
Typical duration of an episode (n = 41)	<24 h	11 (26.8)
	24 h	8 (19.5)
	>24 h	22 (53.7)
Episode intensity	Mild	10 (21.3)
	Moderate	24 (51.1)
	Severe	13 (27.7)

Note. Percentages are of the full sample unless a different denominator is stated.MS subtype, disease-modifying treatment, and disability level (EDSS) were not collected. Self-reported heat-related symptom worsening corresponds to a history of Uhthoff’s phenomenon and was not clinically verified. Characteristics of the interview subgroup (n = 6) are given in Table S3.

**Table 2:** Physical design preferences for the proposed device (n = 47), with 95% Wilson score confidence intervals for the proportion rating each attribute important.

Design Attribute	Important, n (%) (95% CI)	Neutral, n (%)	Less Important, n (%)
Comfortable during prolonged use	34 (72.3) [58.2–83.1]	6 (12.8)	7 (14.9)
Compatible with prescription glasses	31 (66.0) [51.7–77.8]	11 (23.4)	5 (10.6)
Small and discreet design	23 (48.9) [35.3–62.8]	6 (12.8)	18 (38.3)

**Table 3:** Preferred functional features of the proposed device by domain (n = 47), with 95% Wilson score confidence intervals for the proportion rating each feature important. Within each domain, features are listed in descending order of the proportion rating them important for readability only; where confidence intervals overlap, features cannot be distinguished from one another.

Domain	Feature	Important, n (%) (95% CI)	Neutral, n (%)	Less Important, n (%)
**Physiological monitoring**	Monitoring of symptom worsening	42 (89.4) [77.4–95.4]	2 (4.3)	3 (6.4)
	Heart-rate monitoring	36 (76.6) [62.8–86.4]	4 (8.5)	7 (14.9)
	Continuous body-temperature display	35 (74.5) [60.5–84.7]	8 (17.0)	4 (8.5)
	Body-temperature monitoring	32 (68.1) [53.8–79.6]	6 (12.8)	9 (19.1)
**Safety and automated response**	Emergency contact during an attack	40 (85.1) [72.3–92.6]	3 (6.4)	4 (8.5)
	Automatic cooling activation	39 (83.0) [69.9–91.1]	5 (10.6)	3 (6.4)
	Immediate alert (light/sound/vibration)	37 (78.7) [65.1–88.0]	3 (6.4)	7 (14.9)
**Communication and connectivity**	Communication with a healthcare provider	43 (91.5) [80.1–96.6]	1 (2.1)	3 (6.4)
	Integrated health application	41 (87.2) [74.8–94.0]	2 (4.3)	4 (8.5)
	Family access to health status	28 (59.6) [45.3–72.4]	5 (10.6)	14 (29.8)
**Self-management support**	Health reports	44 (93.6) [82.8–97.8]	1 (2.1)	2 (4.3)
	Physical activity tracking	42 (89.4) [77.4–95.4]	4 (8.5)	1 (2.1)
	Symptom recording	41 (87.2) [74.8–94.0]	2 (4.3)	4 (8.5)
	Medication reminders	40 (85.1) [72.3–92.6]	3 (6.4)	4 (8.5)
	Daily symptom diary	38 (80.9) [67.5–89.6]	5 (10.6)	4 (8.5)
	Physiological summaries	36 (76.6) [62.8–86.4]	4 (8.5)	7 (14.9)
	Daily summaries	35 (74.5) [60.5–84.7]	6 (12.8)	6 (12.8)

**Table 4:** Overall acceptability of the proposed device (n = 47), with 95% Wilson score confidence intervals.

Response	n	%	95% CI
Very acceptable	11	23.4	13.6–37.2
Acceptable	5	10.6	4.6–22.6
Somewhat acceptable	6	12.8	6.0–25.2
Neutral	17	36.2	24.0–50.5
Not acceptable	8	17.0	8.9–30.1
Any degree of acceptability (combined)	22	46.8	33.3–60.8
Acceptable or very acceptable (strict definition)	16	34.0	22.2–48.3

**Table 5:** Translation of user priorities into design requirements and verification tests for the next prototype. Percentages are the proportion of the 47 survey participants rating the corresponding item as important.

User Priority (Survey/Interview)	Requirement for the Sensing and Actuation System	Verification Test in the Next Prototype
Comfort during prolonged use (72.3%)	Minimize worn mass and contact pressure at the auricle; place the centre of mass close to the ear axis; select skin-contact materials for multi-hour wear	Contact-pressure mapping; continuous wear trial of at least 4 h with periodic comfort ratings and skin inspection
Compatibility with prescription glasses (66.0%) and with headwear raised in interviews	Geometric clearance at the spectacle temple; retention with hijab or head covering; retention during head movement	Fit trials in spectacle wearers and hijab wearers; measured device displacement during standardized activities of daily living
Monitoring of symptom worsening (89.4%) rated important more often than temperature monitoring (68.1%)	Detect change relative to an individual baseline rather than a fixed absolute threshold; specify thermal resolution, drift and response time; per-user calibration; symptoms captured through user input (symptom recording, daily diary), not measured by the device	Bench characterization of resolution, drift and step response; agreement of the derived thermal index with a reference core-temperature measure (Bland–Altman)
Automatic cooling activation (83.0%); “cooling is more important than just receiving a warning”	Closed-loop actuation with a defined time to onset; airflow sufficient to produce a perceptible change at the lateral neck	Measured airflow velocity at the neck, change in local skin temperature, and thermal sensation votes versus a sham-airflow condition
Immediate multimodal alert (78.7%)	Redundant auditory, tactile, and visual alerting appropriate to co-existing visual and sensory impairment	Alert detectability testing under ambient noise, movement, and with the device worn under a head covering
Emergency contact during an attack (85.1%)	Application-side escalation path with defined latency and a fail-safe when the wireless link drops	End-to-end latency measurement and link-loss testing of the escalation pathway
Health reports (93.6%) and clinician communication (91.5%)	Defined sampling rate, on-device storage, export format and a clinician-facing summary view	Usability testing of the report output with neurologists and rehabilitation clinicians
Family access rated lowest (59.6%)	Granular, per-recipient sharing permissions, disabled by default and controlled by the user	Review of default privacy settings and user testing of the sharing controls
Episodes lasting more than 24 h in 53.7% of respondents	Power budget dominated by the cooling fan; runtime must span a hot day at a realistic duty cycle	Runtime measurement under defined duty cycles; acoustic noise measurement of the fan at the ear
Use in extreme heat: ambient air near or above skin temperature	Ambient-temperature sensing; automatic suspension or limitation of fan operation when air temperature approaches skin temperature; evaluation of hybrid (thermoelectric, evaporative or phase-change) cooling	Climatic-chamber testing at ambient temperatures of 30–45 °C, measuring neck skin temperature and thermal sensation with and without airflow

## Data Availability

The de-identified survey data supporting the conclusions of this article are available from the corresponding author upon reasonable request due to privacy and ethical restrictions.

## Supplementary Materials

The following supporting information can be downloaded at https://www.mdpi.com/article/10.3390/healthcare14203502/s1. Table S1: STROBE checklist for the cross-sectional component; Table S2: COREQ checklist for the qualitative component; Table S3: demographic and clinical characteristics of the full survey sample and the interview subgroup; Table S4a: Inter-item correlations; Table S4b: Spearman inter-item correlation matrix for the 17 functional-feature items; File S1: full survey instrument; File S2: semi-structured interview topic guide.

## Abbreviations

The following abbreviations are used in this manuscript:

CI	Confidence interval
COREQ	Consolidated Criteria for Reporting Qualitative Research
EDSS	Expanded Disability Status Scale
IQR	Interquartile range
mHealth	Mobile health
MS	Multiple sclerosis
PPG	Photoplethysmography
SD	Standard deviation
STROBE	Strengthening the Reporting of Observational Studies in Epidemiology
SUS	System Usability Scale
TAM	Technology Acceptance Model
UTAUT	Unified Theory of Acceptance and Use of Technology
