# Supplementary material for: Perceived Acceptability, Design Preferences, and User Requirements for a Proposed Ear-Worn Device for Heart-Rate and Ear-Temperature Monitoring with Localized Neck Cooling: A Pre-Prototype Mixed-Methods Study in People with Multiple Sclerosis
Source: Healthcare (Basel). 2026 Oct 18;14(20):3502. doi: 10.3390/healthcare14203502 (PMC13704686; doi:10.3390/healthcare14203502)
Supplement: Supplementary file 1 [file healthcare-14-03502-s001.zip › healthcare-4588731-supplementary.pdf]

Supplementary Materials

Table S1. STROBE checklist for the cross-sectional component

Checklist of items that should be included in reports of cross-sectional studies. Section numbers refer to the numbered sections of the manuscript.

Item	Section	Recommendation	Reported in
**1a**	Title and abstract	Indicate the study's design with a commonly used term in the title or the abstract	Title; Abstract
**1b**	Title and abstract	Provide in the abstract an informative and balanced summary of what was done and what was found	Abstract
**2**	Background/rationale	Explain the scientific background and rationale for the investigation being reported	§1, paragraphs 1-5
**3**	Objectives	State specific objectives, including any prespecified hypotheses	§1, final paragraph (three stated aims)
**4**	Study design	Present key elements of study design early in the paper	§2.1
**5**	Setting	Describe the setting, locations, and relevant dates, including periods of recruitment and data collection	§2.3
**6a**	Participants	Give the eligibility criteria, and the sources and methods of selection of participants	§2.3; Figure 2
**7**	Variables	Clearly define all outcomes, exposures, predictors, potential confounders, and effect modifiers	§2.4 (survey domains and items); §2.7
**8**	Data sources / measurement	For each variable of interest, give sources of data and details of methods of assessment; describe comparability of assessment methods if there is more than one group	§2.4; File S1 (full instrument)
**9**	Bias	Describe any efforts to address potential sources of bias	§2.3 (convenience sampling acknowledged); §4.6
**10**	Study size	Explain how the study size was arrived at	§2.3 (no formal sample-size calculation; consecutive eligible respondents in the recruitment window)
**11**	Quantitative variables	Explain how quantitative variables were handled in the analyses; if applicable, describe which groupings were chosen and why	§2.4 (collapsing of the five-point importance scale); §2.7
**12a**	Statistical methods	Describe all statistical methods, including those used to control for confounding	§2.7

**12b**	Statistical methods	Describe any methods used to examine subgroups and interactions	§2.7 (none performed; rationale stated)
**12c**	Statistical methods	Explain how missing data were addressed	§2.7 and §3.1 (item-level denominators stated where they differ, e.g. n = 41 for episode duration)
**12d**	Statistical methods	Describe analytical methods taking account of the sampling strategy	§2.7 (descriptive analysis of a convenience sample)
**12e**	Statistical methods	Describe any sensitivity analyses	Not applicable - no sensitivity analyses were performed
**13a**	Participants	Report numbers of individuals at each stage of the study	§3.1; Figure 2
**13b**	Participants	Give reasons for non-participation at each stage	NA
**13c**	Participants	Consider use of a flow diagram	Figure 2
**14a**	Descriptive data	Give characteristics of study participants and information on exposures and potential confounders	§3.1; Table 1
**14b**	Descriptive data	Indicate the number of participants with missing data for each variable of interest	Table 1 (denominators stated); §3.1
**15**	Outcome data	Report numbers of outcome events or summary measures	§3.2-3.4; Tables 2-4; Figures 3-4
**16a**	Main results	Give unadjusted estimates and, if applicable, confounder-adjusted estimates and their precision	§3.2-3.4 (proportions with 95% Wilson confidence intervals; no adjusted analyses performed)
**16b**	Main results	Report category boundaries when continuous variables were categorized	§2.4 (importance scale collapsed into important / neutral / less important)
**16c**	Main results	If relevant, consider translating estimates of relative risk into absolute risk	Not applicable
**17**	Other analyses	Report other analyses done	§3.5 (qualitative component, reported per COREQ in Table S2)
**18**	Key results	Summarize key results with reference to study objectives	§4.1
**19**	Limitations	Discuss limitations, taking into account sources of potential bias or imprecision	§4.6
**20**	Interpretation	Give a cautious overall interpretation of results	§4.4, §4.5, §5
**21**	Generalizability	Discuss the generalizability (external validity) of the study results	§4.6 (final sentences)
**22**	Funding	Give the source of funding and the role of the funders	Funding statement (back matter)

Table S2. COREQ checklist for the qualitative component

Consolidated criteria for reporting qualitative research: 32-item checklist for interviews and focus groups.

No.	Item	Reported in / response
**Domain 1: Research team and reflexivity**		
**1**	Interviewer / facilitator: Which author conducted the interview or focus group?	*DB*
**2**	Credentials: What were the researcher's credentials?	*BSc*
**3**	Occupation: What was their occupation at the time of the study?	*student researcher*
**4**	Gender: Was the researcher male or female?	*Female*
**5**	Experience and training: What experience or training did the researcher have?	*Got training in qualitative study and interviewing*
**6**	Relationship established: Was a relationship established prior to study commencement?	*No prior relationship; first contact was at recruitment*
**7**	Participant knowledge of the interviewer: What did participants know about the researcher?	*The researcher disclosed - name, role, research purpose*
**8**	Interviewer characteristics: What characteristics were reported about the interviewer?	*interest in the topic & reasons for doing the research*
**Domain 2: Study design**		
**9**	Methodological orientation and theory	§2.7 - reflexive thematic analysis (Braun and Clarke), inductive and semantic coding
**10**	Sampling: How were participants selected?	§2.3 and §2.5 - survey respondents who indicated willingness to be interviewed were invited (see File S1, item 38)
**11**	Method of approach: How were participants approached?	§2.5 - contacted using the telephone number or email address they provided in the survey
**12**	Sample size: How many participants were in the study?	§3.5 - six participants
**13**	Non-participation: How many people refused to participate or dropped out? Reasons?	*0*
**14**	Setting of data collection: Where were the data collected?	§2.5 - conducted remotely (online), at a time chosen by the participant
**15**	Presence of non-participants: Was anyone else present besides the participants and researchers?	*No other person was present*

**16**	Description of sample: What are the important characteristics of the sample?	§3.1 and §3.5
**17**	Interview guide: Were questions, prompts, guides provided by the authors? Was it pilot tested?	File S2 (topic guide) *not pilot tested*
**18**	Repeat interviews: Were repeat interviews carried out?	§2.5 - no repeat interviews were conducted
**19**	Audio/visual recording: Did the research use audio or visual recording to collect the data?	§2.5 - audio-recorded with participant consent
**20**	Field notes: Were field notes made during and/or after the interview?	*No*
**21**	Duration: What was the duration of the interviews?	*40-60 minutes*
**22**	Data saturation: Was data saturation discussed?	§2.5 - saturation was not formally assessed; this is stated as a limitation (§4.6)
**23**	Transcripts returned: Were transcripts returned to participants for comment and/or correction?	*not returned*
**Domain 3: Analysis and findings**		
**24**	Number of data coders: How many data coders coded the data?	§2.7 *2 (DB & MA)*
**25**	Description of the coding tree: Did authors provide a description of the coding tree?	-
**26**	Derivation of themes: Were themes identified in advance or derived from the data?	§2.7 - themes were derived inductively from the data
**27**	Software: What software, if applicable, was used to manage the data?	*Manual coding in Microsoft Word*
**28**	Participant checking: Did participants provide feedback on the findings?	*No*
**29**	Quotations presented: Were participant quotations presented to illustrate the themes?	§3.5 - one illustrative quotation per theme, each identified by participant number
**30**	Data and findings consistent: Was there consistency between the data presented and the findings?	§3.5 and §4 - quotations support each reported theme
**31**	Clarity of major themes: Were major themes clearly presented in the findings?	§3.5 - four themes, each with a heading and narrative summary
**32**	Clarity of minor themes: Is there a description of diverse cases or discussion of minor themes?	§3.5 - divergent accounts reported (e.g. one participant more affected by cold than heat; divergent views on family data sharing)

Table S3. Demographic and clinical characteristics of the full survey sample and the interview subgroup

Values are n (%) unless stated otherwise; percentages are of the column total unless a different denominator is stated. History of Uhthoff's phenomenon was self-reported and not clinically verified. Time since diagnosis was derived from a free-text item. MS subtype, disease-modifying therapy and disability level (EDSS) were not collected. a Derived from the survey export (n = 47); multiple responses were allowed, so percentages do not sum to 100%. Interview participants were identified by linking each transcript to the corresponding survey record.

Characteristic	Category	Full survey sample (N = 47)	Interview subgroup (n = 6)
Age (years)	Mean ± SD (range)	35.6 ± 7.9	36.7 ± 6.4 (29–43)
Sex	Female	29 (61.7)	5 (83.3)
	Male	18 (38.3)	1 (16.7)
Time since diagnosis (years)	Median (IQR) [range]	9 (4–17); n = 45	13.5 (13–17) [5–27]
History of Uhthoff's phenomenon (self-reported heat-related symptom worsening)	Yes	25 (53.2)	3 (50.0)
	Sometimes	15 (31.9)	2 (33.3)
	No	7 (14.9) a	1 (16.7)
Degree of effect of raised body temperature	No effect	3 (6.4)	0
	Mild	10 (21.3)	1 (16.7)
	Moderate	18 (38.3)	2 (33.3)
	Large	16 (34.0)	3 (50.0)
Typical duration of an episode	< 24 h	11 (26.8); n = 41	2 (40.0); n = 5
	24 h	8 (19.5)	0
	> 24 h	22 (53.7)	3 (60.0)
Episode intensity	Mild	10 (21.3)	1 (16.7)
	Moderate	24 (51.1)	2 (33.3)
	Severe	13 (27.7)	3 (50.0)
Heat-related symptoms (multiple responses) a	Severe fatigue	33 (70.2)	5 (83.3)
	General weakness	29 (61.7)	5 (83.3)
	Numbness / tingling	21 (44.7)	2 (33.3)
	Visual problems	17 (36.2)	2 (33.3)

Characteristic	Category	Full survey sample (N = 47)	Interview subgroup (n = 6)
Coping strategies (multiple responses) a	Going to a cool place	25 (53.2)	5 (83.3)
	Stopping the current activity	23 (48.9)	3 (50.0)
	Cooling aids (scarves, vests, ice)	17 (36.2)	4 (66.7)
	Avoiding going out	11 (23.4)	4 (66.7)
Assistive devices used (multiple responses) a	Mobility aid (cane / wheelchair)	26 (55.3)	4 (66.7)
	Prescription glasses	24 (51.1)	4 (66.7)
Overall acceptability rating of the concept	Very acceptable	11 (23.4)	1 (16.7)
	Acceptable	5 (10.6)	0
	Somewhat acceptable	6 (12.8)	1 (16.7)
	Neutral	17 (36.2)	2 (33.3)
	Not acceptable	8 (17.0)	2 (33.3)
MS subtype, disease-modifying therapy, EDSS	—	Not collected	Not collected

Table S4.a Inter-item correlations

Its summarized by a priori domain for the 17 functional-feature items (Spearman correlations of the original five-point ratings, N = 47). Family access to health status (C3) correlated weakly with all other items (ϱ = −0.12 to 0.22). Exploratory factor analysis was not performed because the sample (fewer than three respondents per item) is below commonly recommended minimums.

Domain (a priori)	Items	Mean within-domain ϱ (range)	Cronbach's α	Mean ϱ with items of other domains
Physiological monitoring	M1–M4	0.61 (0.41–0.81)	0.86	0.37
Safety and automated response	S1–S3	0.39 (0.19–0.61)	0.60	0.40
Communication and connectivity	C1–C3	0.30 (0.08–0.67)	0.42	0.37
Self-management support	H1–H7	0.56 (0.36–0.73)	0.85	0.40
All 17 items	—	—	0.89	Mean between-domain ϱ = 0.39

Table S4.b Spearman inter-item correlation matrix for the 17 functional-feature items (N = 47)

M = physiological monitoring; S = safety and automated response; C = communication and connectivity; H = self-management support.

Item	M1	M2	M3	M4	S1	S2	S3	C1	C2	C3	H1	H2	H3	H4	H5	H6	H7
M1 Monitoring of symptom worsening	1																
M2 Heart-rate monitoring	0.52	1															
M3 Continuous temperature display	0.41	0.77	1														
M4 Temperature monitoring	0.47	0.68	0.81	1													
S1 Emergency contact	0.47	0.07	0.00	−0.06	1												
S2 Automatic cooling activation	0.41	0.45	0.53	0.51	0.38	1											
S3 Immediate alert	0.35	0.51	0.54	0.68	0.19	0.61	1										
C1 Communication with healthcare provider	0.71	0.40	0.32	0.25	0.75	0.58	0.38	1									
C2 Integrated health application	0.79	0.60	0.49	0.52	0.44	0.50	0.42	0.67	1								
C3 Family access	0.06	0.11	−0.12	−0.03	0.22	−0.03	0.13	0.14	0.08	1							
H1 Health reports	0.72	0.25	0.17	0.17	0.52	0.28	0.24	0.66	0.58	0.13	1						
H2 Physical activity tracking	0.57	0.32	0.19	0.34	0.46	0.34	0.27	0.50	0.57	0.09	0.68	1					
H3 Symptom recording	0.48	0.38	0.27	0.41	0.45	0.41	0.47	0.58	0.48	0.06	0.64	0.73	1				
H4 Medication reminders	0.59	0.31	0.23	0.23	0.56	0.44	0.26	0.76	0.64	0.09	0.72	0.65	0.62	1			
H5 Daily symptom diary	0.31	0.20	0.27	0.31	0.55	0.51	0.54	0.55	0.38	0.08	0.48	0.38	0.68	0.58	1		
H6 Physiological summaries	0.48	0.53	0.55	0.64	0.29	0.54	0.69	0.45	0.43	0.01	0.36	0.40	0.52	0.48	0.62	1	
H7 Daily summaries	0.52	0.41	0.48	0.58	0.15	0.28	0.60	0.36	0.37	0.17	0.58	0.42	0.62	0.46	0.54	0.69	1

File S1. Full survey instrument

The survey was delivered in Arabic and was open from 11 February 2026 to 17 April 2026. Median completion time in the exported response file was approximately 7 minutes (range 2-23 min).

Scale coding. Two ordinal scales were used, both numbered by the form itself. Importance: (1) not important, (2) somewhat important, (3) neutral, (4) important, (5) very important. Acceptability: (1) not acceptable, (2) somewhat acceptable, (3) neutral, (4) acceptable, (5) very acceptable. Note that on both scales the form placed the 'somewhat' option below 'neutral'. For analysis, importance responses were collapsed as important = (4) + (5), neutral = (3), and less important = (1) + (2).

No.	Item (Arabic, as administered)	English translation	Response options
**Section 1. Consent**			
1	هل توافق على المشاركة بالدراسة؟	Do you agree to take part in this study?	نعم / لا<br>Yes / No
**Section 2. Demographic and clinical characteristics**			
2	الجنس	Sex	ذكر / أنثى<br>Male / Female
3	العمر	Age	Free text (years)
4	منذ متى تم تشخيصك؟	How long ago were you diagnosed?	Free text
**Section 3. Heat-related symptom worsening**			
5	هل تعاني من تفاقم الأعراض عند ارتفاع درجة الحرارة؟	Do you experience worsening of your symptoms when your body temperature rises?	نعم / أحيانًا / لا<br>Yes / Sometimes / No
6	ما مدى تأثرك بارتفاع درجة حرارة الجسم؟	To what extent are you affected by a rise in body temperature?	لا يؤثر / تأثير بسيط / تأثير متوسط / تأثير شديد<br>No effect / Mild / Moderate / Large
7	كم تستغرق مدة الهجمة عادة؟	How long does an episode usually last?	أقل من 24 ساعة / حوالي 24 ساعة / أكثر من 24 ساعة<br>Less than 24 h / About 24 h / More than 24 h
8	كم مرة تحدث لك هذه الهجمات عادة؟	How often do these episodes usually occur?	يوميًا / أسبوعيًا / شهريًا / سنويًا<br>Daily / Weekly / Monthly / Yearly
9	بناءً على إجابتك السابقة، في حال تذكرك اذكر كم عدد الهجمات؟	Based on your previous answer, if you can recall, how many episodes have you had?	Free text
10	كيف تصف شدة الهجمة التي تعاني منها؟	How would you describe the severity of the episodes you experience?	خفيفة: أعراض بسيطة لا تعيق الأنشطة اليومية / متوسطة: أعراض تؤثر على بعض الأنشطة اليومية / شديدة: أعراض تؤثر بشكل كبير على القدرة على أداء الأنشطة اليومية<br>Mild: minor symptoms that do not impede daily activities / Moderate: symptoms that affect some daily activities / Severe: symptoms that substantially affect the ability to perform daily activities
11	ما أكثر الأعراض التي تظهر عليك عند ارتفاع الحرارة؟ (يمكن اختيار أكثر من خيار)	Which symptoms most commonly appear when your temperature rises? (multiple selection allowed)	إرهاق شديد / ضعف عام / خدر أو تنميل / مشاكل بالرؤية / أخرى (يرجى التحديد)<br>Severe fatigue / General weakness / Numbness or tingling / Visual problems / Other (please specify)
12	متى تلاحظ الأعراض عادة؟	When do you usually notice the symptoms?	في المنزل / أثناء الخروج / أثناء النشاط البدني / لا أستطيع التحديد<br>At home / While outdoors / During physical activity / Cannot determine

**13**	في حال ارتفاع درجة حرارة الجسم، كيف تتعامل مع الهجمة؟ (يمكن اختيار أكثر من إجابة)	When your body temperature rises, how do you manage the episode? (multiple selection allowed)	الذهاب إلى مكان بارد / التوقف عن النشاط الذي كنت تقوم به / استخدام وسائل تبريد (أوشحة، سترات، ثلج) / تجنب الخروج / لا أستخدم أي وسيلة / أخرى<br>Move to a cool place / Stop the current activity / Use cooling aids (scarves, vests, ice) / Avoid going out / I do not use any method / Other
**14**	ما مدى رضاك عن هذه الحلول؟	How satisfied are you with these solutions?	غير راضٍ / راضٍ إلى حد ما / راضٍ<br>Not satisfied / Somewhat satisfied / Satisfied
**15**	هل استخدمت أيًا من الأجهزة التالية؟ (يمكن اختيار أكثر من إجابة)	Have you used any of the following devices? (multiple selection allowed)	نظارة طبية / أجهزة مساعدة للحركة (عكاز أو كرسي متحرك) / جهاز اهتزاز / لا شيء / أخرى<br>Prescription glasses / Mobility aids (cane or wheelchair) / Vibration device / None / Other
**Section 4. Overall acceptability of the proposed device**			
**16**	ما مدى قبولك لفكرة جهاز يُرتدى داخل الأذن؟	How acceptable do you find the idea of a device worn in the ear?	غير مقبول (1) / مقبول إلى حد ما (2) / محايد (3) / مقبول (4) / مقبول جدًا (5)<br>Not acceptable (1) / Somewhat acceptable (2) / Neutral (3) / Acceptable (4) / Very acceptable (5)
**Section 5. Physical design preferences**			
**17**	هل يهمك أن يكون الجهاز صغير الحجم ؟	Is it important to you that the device is *small*?	Importance scale (1)-(5)
**18**	هل يهمك أن يكون الجهاز مريحًا عند الاستخدام؟	Is it important to you that the device is comfortable in use?	Importance scale (1)-(5)
**19**	هل يهمك أن يكون الجهاز متوافقًا مع النظارة؟	Is it important to you that the device is compatible with glasses?	Importance scale (1)-(5)
**Section 6. Functional feature priorities**			
**20**	ما مدى أهمية وجود تطبيق يراقب تفاقم الأعراض لديك؟	How important is an application that monitors the worsening of your symptoms?	Importance scale (1)-(5)
**21**	ما مدى أهمية تفضيلك لوجود جميع بياناتك الصحية في تطبيق واحد؟	How important is it to you to have all of your health data in a single application?	Importance scale (1)-(5)
**22**	ما مدى أهمية مراقبة درجة حرارة الجسم لمرضى التصلب اللويحي؟	How important is monitoring of body temperature for people with multiple sclerosis?	Importance scale (1)-(5)
**23**	ما مدى أهمية عرض درجة حرارة الجسم بشكل مباشر في التطبيق طوال اليوم؟	How important is a live display of body temperature in the application throughout the day?	Importance scale (1)-(5)
**24**	ما مدى أهمية متابعة نبضات القلب عبر التطبيق؟	How important is heart-rate monitoring through the application?	Importance scale (1)-(5)
**25**	ما مدى أهمية التنبيه الفوري عند ارتفاع درجة الحرارة (ضوء أو صوت أو اهتزاز)؟	How important is an immediate alert when your temperature rises (light, sound or vibration)?	Importance scale (1)-(5)
**26**	ما مدى أهمية وجود تشغيل تلقائي لنظام التبريد يساعد في تبريد الجسم؟	How important is automatic activation of a cooling system that helps to cool the body?	Importance scale (1)-(5)
**27**	في حال حدوث هجمة، ما مدى أهمية وجود خيار الاتصال بالطوارئ؟	In the event of an episode, how important is having an emergency contact option?	Importance scale (1)-(5)

**28**	ما مدى أهمية وجود خيار التواصل مع مقدم الرعاية الطبية عبر التطبيق؟	How important is the option to communicate with your healthcare provider through the application?	Importance scale (1)-(5)
**29**	ما مدى أهمية تمكين العائلة من الاطلاع على حالتك الصحية عبر التطبيق؟	How important is enabling your family to view your health status through the application?	Importance scale (1)-(5)
**30**	ما مدى أهمية وجود مفكرة يومية لتسجيل الأعراض داخل التطبيق؟	How important is a daily diary for recording symptoms within the application?	Importance scale (1)-(5)
**31**	ما مدى أهمية وجود تذكير بالأدوية أو مواعيد العلاج؟	How important are reminders for medication or treatment appointments?	Importance scale (1)-(5)
**32**	ما مدى أهمية تتبع النشاط البدني والتمارين عبر التطبيق؟	How important is tracking of physical activity and exercise through the application?	Importance scale (1)-(5)
**33**	ما مدى أهمية تدوين الأعراض لمساعدة الطبيب أو المعالج على فهم حالتك بشكل أفضل؟	How important is recording symptoms to help your doctor or therapist understand your condition better?	Importance scale (1)-(5)
**34**	ما مدى أهمية الاطلاع على التقارير الصحية عبر التطبيق؟	How important is access to health reports through the application?	Importance scale (1)-(5)
**35**	ما مدى أهمية وجود ملخصات يومية عبر التطبيق؟	How important are daily summaries within the application?	Importance scale (1)-(5)
**36**	ما مدى أهمية وجود ملخص لحالات (ارتفاع درجة الحرارة، معدل ضربات القلب، ...)؟	How important is a summary of episodes (temperature rise, heart rate, and similar)?	Importance scale (1)-(5)
**37**	هل ترى أن السماعة الذكية، من حيث شكلها وخفة وزنها، تساهم في تحسين راحتك؟	Do you consider that the shape and light weight of the smart earbud would improve your comfort?	Importance scale (1)-(5)
**Section 7. Invitation to interview**			
**38**	هل ترغب بالمشاركة في مقابلة بحثية عن تجربتك الشخصية، حيث ستكون عن بُعد (أونلاين) بالوقت المناسب لك؟	Would you like to take part in a research interview about your personal experience? It would be conducted remotely (online) at a time convenient for you.	نعم / لا<br>Yes / No
**39**	يرجى كتابة رقم التواصل	Please write your contact number	Free text (collected only from participants who agreed to be interviewed)
**40**	يرجى كتابة الإيميل	Please write your email address	Free text (collected only from participants who agreed to be interviewed)

File S2. Semi-structured interview topic guide

Interviews were conducted remotely in Arabic with survey respondents who had indicated willingness to be contacted (item 38 of File S1). The guide below is semi-structured: the numbered questions are the core prompts and the indented items are probes used where relevant. Interviewers were free to follow the participant's account and to change the order of questions. The guide is reproduced in English with the Arabic wording used in the interviews.

Before beginning, the interviewer restated the purpose of the study, confirmed verbal consent for audio recording, reminded the participant that they could stop at any time, and confirmed that no identifying information would appear in any report.

No.	Question / probe (English)	Arabic wording
**Part 1. Living with multiple sclerosis**		
1	To start, tell me about your experience with multiple sclerosis, from your diagnosis until now.	بدايةً، حدثني عن تجربتك مع التصلب اللويحي منذ التشخيص حتى الآن.
	Probe: When and how were you diagnosed? What were the first symptoms you noticed?	استفسار: متى وكيف تم التشخيص؟ وما أول الأعراض التي لاحظتها؟
2	What was the hardest thing for you at the beginning, at the time of diagnosis?	ما أصعب شيء واجهته في البداية، عند التشخيص؟
3	Apart from spasticity, what other symptoms do you get because of the disease?	بعيدًا عن التشنج، ما الأعراض الأخرى التي تظهر عليك بسبب المرض؟
**Part 2. Episodes and their impact**		
4	Tell me about the episodes you experience - what happens and how do they start?	حدثني عن الهجمات التي تتعرض لها؛ ماذا يحدث فيها وكيف تبدأ؟
5	How do these episodes affect your daily activities?	كيف تؤثر هذه الهجمات على أنشطتك اليومية؟
	Probe: Is there anything you stop doing, or avoid, during an episode?	استفسار: هل هناك أشياء تتوقف عنها أو تتجنبها أثناء الهجمة؟
6	Has the frequency of the episodes changed over time?	هل تغير عدد الهجمات أو تكرارها مع مرور الوقت؟
7	How do you tell the difference between a real relapse and a temporary worsening of symptoms?	كيف تفرّق بين الهجمة الحقيقية والتدهور المؤقت في الأعراض؟
**Part 3. Heat and its management**		
8	Does a rise in temperature affect you?	هل يؤثر عليك ارتفاع درجة الحرارة؟
9	Have you ever cancelled or changed a plan because of the heat?	هل سبق أن ألغيت أو غيّرت خططك بسبب الحرارة؟
10	Do your symptoms increase when you go out in the sun or in hot weather?	هل تزداد الأعراض عند الخروج في الشمس أو في الجو الحار؟
11	What do you do when you feel that your temperature has risen?	ماذا تفعل عندما تشعر بارتفاع درجة حرارتك؟
	Probe: air conditioning, cold water, cooling aids, resting, stopping the activity?	استفسار: التكييف، الماء البارد، وسائل التبريد، الراحة، التوقف عن النشاط؟
12	How well do these methods work for you?	إلى أي مدى تنجح هذه الوسائل معك؟
**Part 4. Reaction to the proposed device**		

**13**	The device we are working on relates to temperature. It is an earpiece worn on the ear that monitors your temperature and heart rate; if your temperature rises it sends you an alert, and an automatic cooling unit directs air towards the neck area. What do you think of this idea?	الجهاز الذي نعمل عليه يتعلق بدرجة الحرارة. وهو سماعة تُلبس على الأذن تراقب درجة الحرارة ومعدل نبضات القلب، وعند ارتفاع الحرارة ترسل لك تنبيهًا، ويوجد نظام تبريد تلقائي يوجّه الهواء نحو منطقة الرقبة. ما رأيك في هذه الفكرة؟
**14**	Is wearing it on or inside the ear socially acceptable for you? And would it work alongside glasses, given that many people with MS have vision problems?	هل ترى أن ارتداءه على الأذن أو داخلها مقبول اجتماعيًا بالنسبة لك؟ وهل يتوافق مع النظارة، خاصة أن كثيرًا من مرضى التصلب اللويحي لديهم مشاكل في الرؤية؟
	Probe: appearance, size, weight, wearing it with a head covering, wearing it for several hours.	استفسار: الشكل، الحجم، الوزن، ارتداؤه مع غطاء الرأس، ارتداؤه لعدة ساعات.
**15**	The earpiece connects to an application. Would improvements to the application be useful to you? Do you have any concerns about the privacy of the information it holds?	السماعة مرتبطة بتطبيق. هل ترى أن تطوير التطبيق مفيد لك؟ وهل لديك أي تحفظ على خصوصية المعلومات التي يحتويها؟
**16**	The application could let your doctor review when the last episode was, how long it lasted and what happened. What do you think about that?	يمكن للتطبيق أن يتيح لطبيبك الاطلاع على وقت آخر هجمة ومدتها وما حدث فيها. ما رأيك في ذلك؟
	Probe: who else should be able to see the information - family, carers? Who should not?	استفسار: من غيرك يمكنه الاطلاع على المعلومات؛ العائلة، مقدمو الرعاية؟ ومن لا ينبغي أن يطلع عليها؟
**17**	If a device like this existed, would the alert on its own be enough, or is the cooling the more important part?	لو وُجد جهاز مثل هذا، هل يكفي التنبيه وحده، أم أن التبريد هو الجزء الأهم؟
**Part 5. Recommendations and closing**		
**18**	What final recommendations would you give us if we design a device like this?	ما توصياتك النهائية لنا إذا صممنا جهازًا مثل هذا؟
**19**	Is there anything I did not ask you about that is important regarding the disease, or an improvement you would want?	هل هناك شيء لم أسألك عنه وتراه مهمًا بخصوص المرض، أو تحسين ترغب فيه؟
**20**	In your view, what does a person with multiple sclerosis most need?	برأيك، ما أكثر ما يحتاجه المصاب بالتصلب اللويحي؟
	Closing: thank the participant, confirm that the recording will be transcribed and anonymised, and ask whether they wish to receive a summary of the findings.	الختام: شكر المشارك، والتأكيد على أن التسجيل سيُفرّغ ويُجهّل، وسؤاله إن كان يرغب في الحصول على ملخص النتائج.
